# Novel paradigm of mosquito-borne disease control based on self-powered strategy

**DOI:** 10.3389/fpubh.2023.1115000

**Published:** 2023-01-20

**Authors:** Junhao Wang, Zhiyuan Zhu

**Affiliations:** ^1^School of Electronic Information Engineering, Southwest University, Chongqing, China; ^2^State Key Laboratory of Bioelectronics, Southeast University, Nanjing, China

**Keywords:** mosquito-borne, public health, self-powered, triboelectric nanogenerators, nano energy

## Introduction

Mosquito is an ancient creature, which can be traced back to the age of dinosaurs (1). There are more than 3,500 species of mosquitoes, in which *Aedes, Anopheles* and *Culex* bear primary responsibility for the spread of human diseases (2). Because of their small size and strong adaptability, mosquitoes can transmit bacteria, viruses, or parasites, which threaten >40% of the world's population. Thus mosquitoes are becoming an increasingly serious global public health challenge (3). When mosquitoes carrying pathogens bite people, they inject the pathogens into the human skin through a suction mouthpiece, following which the pathogens attack the subcutaneous immune cells at the bite site (4). The pathogens are amplified in these cells and released into the blood, causing systemic infection, and the symptoms include fever, encephalitis, arthritis, and hemorrhagic fever. However, mosquitoes transmit diseases without being affected. Although recent studies have shown that mosquitoes do not transmit the COVID-19 virus, the COVID-19 global pandemic is hampering mosquito-borne disease control efforts, such as disrupting malaria services (5). This not only increases the risk of mosquito-borne pathogens transmission but also lead to a significant increase in the number of cases and deaths (6, 7). According to the statistics of the World Health Organization, more than 700,000 deaths caused by mosquito-borne diseases annually (8). In fact, not only humans, but most other land mammals are also victims of mosquito bites (9). Therefore, accelerating technical research on the prevention of these mosquito-borne diseases can prevent unnecessary deaths.

Despite the efforts of all countries in preventing mosquito-borne diseases, achieving effective and sustainable mosquito control is still challenging (10, 11). Efficient sterilization and mosquito control are required to significantly reduce the risk of disease transmission and guarantee restrategy of public health problems. Currently, chemical and physical methods of killing mosquitoes are used, although they have obvious shortcomings. Long-term use of chemical methods is harmful to the human body; in addition, chemical reagents pollute the environment (12, 13). In contrast, although the physical methods are reliable, their use is associated with more limitations; for example, high-voltage electricity is dangerous and it relies on a continuous power supply (14–16).

The triboelectric nanogenerator (TENG) is a new technology based on electrification of mechanical interface friction and electrostatic induction coupling effect. The advantages of TENG include a wide selection of materials, simple structure, low cost, high efficiency, and high output voltage (17). It has also been widely used in fields involving motion (18), pressure (19, 20), inertia (21) and vibration (22, 23) to convert mechanical energy to electrical energy; furthermore, it can also be used in conjunction with wind energy (24), water energy (21, 25), and other fields (26, 27). As most mosquito control equipment require external power supply, the limitations of mosquito control and sterilization are manifold, especially in outdoor and remote areas. TENG technology is effective for integrated self-powered equipment. Therefore, development of an environment-friendly and maintenance-free control equipment for sterilization and infection is necessary.

**Figure 1:**
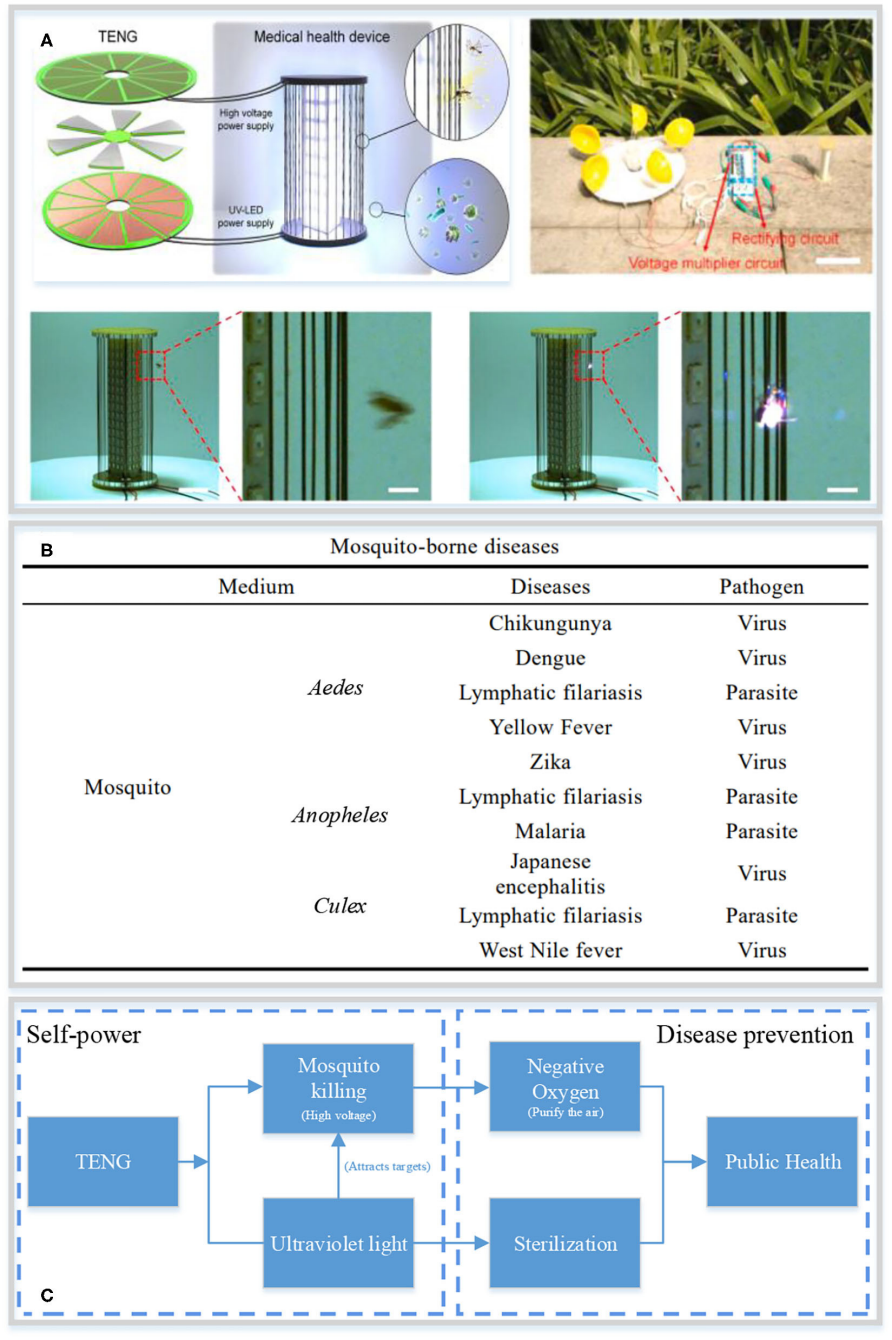
The new paradigm of mosquito-borne disease control. **(A)** Structural design and photographs of the TENG-based self-powered sterilization and infection control system (28). **(B)** Mosquito-borne diseases. **(C)** Strategy for mosquito-borne disease prevention based on self-powered supply.

Here, we envisage a self-powered strategy for sterilization and infection control based on TENG structure, which can be used for mosquito eradication and sterilization to control the number of mosquitoes and the pathogens they transmit, thereby reducing the risk of disease transmission (Figure 1A). This strategy will open up a new avenue for self-powered supply system in infectious disease control and may promote family health care and public health.

## Analysis

### Epidemic status of mosquito-borne diseases

Mosquito-borne diseases are natural foci diseases transmitted by vector mosquitoes, which mainly include *Aedes, Anopheles*, and *Culex* mosquitoes (Figure 1B). *Aedes* is distributed worldwide, especially in the tropics and subtropics. It is the largest genus of mosquitoes, including 38 subgenera and nearly 1,000 species, and is the main vector of urban yellow fever (29), chikungunya virus (30) and dengue fever (31, 32). Experimental infection models have shown that it can also transmit Venezuelan equine encephalitis, western equine encephalitis, eastern equine encephalitis, rift valley fever,zika virus, and other viruses by biting and sucking blood (33, 34). Thus, it is one of the most dangerous mosquitoes. Reports show that the dengue virus transmitted by *Aedes* mosquitoes causes nearly 96 million symptomatic cases and 40,000 deaths every year. At the same time, more than 3.9 billion people in 129 countries are still at risk of contracting dengue fever (35).

The female *Anopheles* mosquito, also known as the malaria mosquito, transmits malaria and filaria to humans. About 450 species of *Anopheles* are known, most of which are distributed in the tropics, mainly in Saharan Africa. Malaria is a life threatening disease caused by the *Plasmodium* parasites, which are transmitted to humans through *Anopheles* bites (36). In early days, the disease caused 0.725 to 1 million deaths every year, which set the Guinness World Record and was also called the deadliest disease on earth. According to the latest World Malaria Report (37), there were 241 million cases of malaria in 2020 and 227 million cases in 2019. Although malaria is preventable and treatable, an increase in the resistance of mosquitoes to insecticides hampers the effectiveness of preventive measures (38).

*Culex* mosquitoes transmit pathogens causing filaria, Japanese encephalitis, and other diseases. Lymphatic filariasis affects the host's lymphatic system, subcutaneous tissue, abdominal cavity, chest cavity, and other places (39). In total, 863 million people in 47 countries are still threatened by lymphatic filariasis, and preventive chemotherapy is required to prevent the transmission of this parasitic infection (40). However, Japanese encephalitis also poses a threat to public safety.

Today, despite the continuous development of medical technology, pathogens still infect more than one million people every year, posing a growing threat to public health. This is amplified further by the lack of effective treatment methods for most infectious diseases and the accumulation of mutations in viruses, which should be tracked for early prevention and interruption of transmission.

### Strategy for mosquito-borne disease prevention based on self-powered supply

#### Basis of self-powered supply system based on TENG

Currently, TENG is widely used in wearable electronics, the Internet of Things, high-precision sensors, and other fields (41). The main reasons for the large-scale use of TENG are as follows: first, TENG allows extensive material selection and is inexpensive (42). Its performance depends on the materials used to develop them, as the friction charges and electrical properties differ with materials. According to the principle of potential superposition, the output voltage and current are affected by the density of friction charges. Therefore, the materials used to develop TENG must easily generate friction charges and have different friction electrodes. TENG is usually made of polytetrafluoroethylene (PTFE) (43), polyamide, polyvinylidene fluoride (PVDF) (44), and silk (45).

Second, TENG has the advantages of simple structure, high efficiency, and high output voltage. The four basic working modes of TENG are vertical contact separation mode, horizontal sliding mode, single electrode mode, and independent layer mode (46). In the simplest design of TENG, the dielectric films of two different materials are stacked face to face, and their respective back surfaces are coated with metal electrodes in the vertical contact separation mode structure. These two layers of dielectric films contact each other to form opposite charges. An induced potential difference is formed between the two electrodes when the films are separated, which causes the current to flow between the connected metal electrodes. The typical high voltage outputs of TENG can exceed 2 kV for single electrode TENG, 15 kV for independent layer TENG, 7 kV for contact separated TENG and 8 kV for disk TENG, respectively (47).

Third, TENG can be integrated with other systems to realize different types of self-powered microsystems, build a functional and structural integrated microsystem of micro mechanical energy collection management storage utilization, and provide a sustainable power supply for micro-electro-mechanical system (MEMS) devices and micro integrated systems in different environments. TENG not only collects exercise energy, but also powers wearable electronic devices. It is also used to collect the energy generated from life activities, such as heartbeat and breathing, and to provide power for *in vitro* monitoring devices and implanted electronic medical devices for disease treatment and prevention. The realization of self-powered supply based on TENG equipment has more advantages than the existing mosquito-borne disease prevention technologies.

#### Mosquito killing and sterilization strategy based on self-powered supply

Currently, preventing the spread of infectious diseases at the prevention stage, which will safeguard the public considerably, is the most critical issue that requires attention. The TENG technology can be integrated into sensor devices (48, 49), wearable electronic devices (50) and medical devices (51) to realize self-powered supply function. Therefore, the requirement of external power drives and sterilization after mosquito control should be met.

On the one hand, though the voltage output of TENG is high, the current of TENG is very low (52–54), which is safe for people (safe current < 30 mA).^1^ On the contrary, as mosquitoes are very small, their endurance is considerably much lower than that of human beings, which can be killed by TENG outputs [as has been demonstrated by Luo et al. (28)]. For conventional high-voltage mosquito-killing device, external power sources are indispensable for driving the relevant devices, which will lead to risk such as power Gide electricity leakage, and fire risk. For instance, electric mosquito swatters are blamed for causing fire.^2^ In comparison, TENG can convert low frequency, distributed, and irregular mechanical energy into electricity, which do not need external power sources. This can reduce the risk of electricity leakage and fire during electricity transmission from the power Gride.

On the other hand, appropriate light sources can be selected to trap mosquitoes according to their phototactic habits. According to a survey, mosquitoes tend to gather near light sources in the ultraviolet and blue ultraviolet wave bands, especially at 365, 405, 420, and 450 nm, among which the sources emitting light at 365 and 420 nm show the most outstanding mosquito-trapping performance, with no significant difference between the effects of the two (16, 55). Therefore, appropriate ultraviolet light source can be selected to trap mosquitoes using electric shock.

Hence, the self-powered tool is harmless and safe for the human body and can be used as a family-level tool. More importantly, the mosquito killing and sterilization system based on TENG can not only present a self-powered working strategy, but can also achieve the goal of purifying air with high-pressure ionized oxygen anion, killing bacteria using ultraviolet radiation and controlling the spread of diseases (Figure 1C).

## Discussion

The performance of TENG is the basis for long-term stable operation of a self-powered equipment. However, realizing the stable application of TENG is challenging. Ideally, only when the mosquito-borne disease prevention technology is stable and reliable can these interventions help to control the spread of infections and minimize the damage to society and economy. To promote this, ongoing research should focus on the following areas.

The self-powered equipment should collect micro mechanical energy in various environments, such as wind energy, water energy, and tidal energy. At the same time, efficient mosquito control requires higher output voltage and energy. The output voltage, energy, and charging cycle are the key parameters required to obtain better mosquito control. Therefore, collisions caused by the natural environment should be prevented, and the output performance should be reliable and stable.

The four working modes of TENG depend on the contact friction of different materials. However, the contact friction will lead to heat loss and wear and tear of the friction interface. The surface charge density of TENG will decrease with increase in its service time, affecting its output performance. Therefore, in the future, means of avoiding device wear to achieve more extensive and effective micro energy collection and application should be investigated.^2^

Enriching the diversity of prevention measures and improving the effectiveness of disease prevention is critical for preventing and controlling the outbreak and transmission of mosquito-borne virus diseases (28). Therefore, assessing the reliability and practicality of new prevention means is extremely important. In this context, a TENG with high durability and high output performance should be developed to realize a self-powered strategy for mosquito-borne disease control.

## Conclusion

This paper discusses the epidemic situation and trend in the development of mosquito-borne infectious diseases. It also analyzes the theoretical basis of TENG as a self-powered equipment and discusses its potential as a strategy to mosquito-borne diseases based on TENG structure. In the future, with the continuous development of TENG technology, the self-powered mosquito-borne disease control strategy will also be improved gradually, especially in outdoor and remote areas. The environmental protection and maintenance-free sterilization and infection control strategy are important for public safety.

## Author contributions

JW: writing the paper. ZZ: supervised. Both authors contributed to the article and approved the submitted version.

## Footnotes

^1^ https://www.d.umn.edu/~sburns/EE2212/L-Safe-Levels-of-Current-in-the-Human-Body.pdf

^2^ https://asiatimes.com/2018/04/electric-mosquito-swatter-blamed-causing-fire/

## References

[1] Shoujun L Zhiping W. Correlation between evolution of biosphere and earth shell evolution. J China Med Univ. 04:1998.

[2] Ruzzante L Reijnders MJMF Waterhouse RM. Of genes and genomes: mosquito evolution and diversity. Trends Parasitol. (2019) 35:32–51. 10.1016/j.pt.2018.10.003 30391118

[3] Chala B Hamde Emerging F. Re-emerging vector-borne infectious diseases and the challenges for control: a review. Front Public Health. (2021) 9:715759. 10.3389/fpubh.2021.715759 34676194 PMC8524040

[4] Thongsripong P Hyman JM Kapan DD Bennett SN. Human-mosquito contact: a missing link in our understanding of mosquito-borne disease transmission dynamics. Ann Entomol Soc Am. (2021) 114:397–414. 10.1093/aesa/saab011 34249219 PMC8266639

[5] Huang YS Vanlandingham DL Bilyeu AN Poramathikul K Harncharoenkul K Kuntawunginn W. SARS-CoV-2 failure to infect or replicate in mosquitoes: an extreme challenge. Sci Rep. (2020) 10:11915. 10.1038/s41598-020-68882-7 32681089 PMC7368071

[6] Elaiw AM Al Agha AD. Global Stability of a reaction–diffusion malaria/COVID-19 coinfection dynamics model. Mathematics. (2022) 10:4390. 10.3390/math10224390

[7] Boonyarangka P Phontham K Sriwichai S Poramathikul K Harncharoenkul K Kuntawunginn W. Co-Infection with Plasmodium vivax and COVID-19 in Thailand. Trop Med Infect Dis. (2022) 7:145. 10.3390/tropicalmed7080145 35893653 PMC9332623

[8] WHO-World Health Organization. Vector-Borne Diseases. (2020). Available online at: https://www.who.int/news-room/fact-sheets/detail/vector-borne-diseases.

[9] Mubemba B Mburu MM Changula K Muleya W Moonga LC Chambaro HM. Current knowledge of vector-borne zoonotic pathogens in Zambia: a clarion call to scaling-up “One Health” research in the wake of emerging and re-emerging infectious diseases. PLoS Negl Trop Dis. (2022) 16:e0010193. 10.1371/journal.pntd.0010193 35120135 PMC8849493

[10] Maciel-de-Freitas R Avendanho FC Santos R Sylvestre G Araújo SC Lima JBP. Undesirable consequences of insecticide resistance following Aedes aegypti control activities due to a dengue outbreak. PLoS ONE. (2014) 9:e92424. 10.1371/journal.pone.0092424 24676277 PMC3968006

[11] McGraw EA O'Neill SL. Beyond insecticides: new thinking on an ancient problem. Nat Rev Microbiol. (2013) 11:181–93. 10.1038/nrmicro2968 23411863

[12] Jahir A Kahamba NF Knols TO Jackson G Patty NFA Shivdasani S. Mass trapping and larval source management for mosquito elimination on small Maldivian islands. Insects. (2022) 13:805. 10.3390/insects13090805 36135506 PMC9503984

[13] Adhikari K Khanikor B Sarma R. Persistent susceptibility of Aedes aegypti to eugenol. Sci Rep. (2022) 12:2277. 10.1038/s41598-022-06302-8 35145175 PMC8831528

[14] Li Y Su X Zhou G Zhang H Puthiyakunnon S Shuai S. Comparative evaluation of the efficiency of the BG-Sentinel trap, CDC light trap and Mosquito-oviposition trap for the surveillance of vector mosquitoes. Parasit Vectors. (2016) 9:446. 10.1186/s13071-016-1724-x 27519419 PMC4983048

[15] Sriwichai P Karl S Samung Y Sumruayphol S Kiattibutr K Payakkapol A. Evaluation of CDC light traps for mosquito surveillance in a malaria endemic area on the Thai-Myanmar border. Parasit Vectors. (2015) 8:636. 10.1186/s13071-015-1225-3 26666683 PMC4678759

[16] Mwanga EP Ngowo HS Mapua SA Mmbando AS Kaindoa EW Kifungo K.. Evaluation of an ultraviolet LED trap for catching Anopheles and Culex mosquitoes in south-eastern Tanzania. Parasites Vectors. (2019) 12:418. 10.1186/s13071-019-3673-7 31455370 PMC6712696

[17] Bai Y Feng H Li Z. Theory and applications of high-voltage triboelectric nanogenerators, *Cell Reports Phys Sci*. (2022) 3:101108. 10.1016/j.xcrp.2022.101108

[18] Zhang Z Cai J High output triboelectric nanogenerator based on PTFE and cotton for energy harvester and human motion sensor. Curr Appl Phys. (2021) 22:1–5. 10.1016/j.cap.2020.11.001

[19] Wang ZL Song J. Piezoelectric nanogenerators based on zinc oxide nanowire arrays. Science. (2006) 312:242–6. 10.1126/science.1124005 16614215

[20] Xu Q. Wen J, Qin Y. Development and outlook of high output piezoelectric nanogenerators. Nano Energy. (2021) 86:106080. 10.1016/j.nanoen.2021.106080

[21] Kim T Chung J Kim D Moon J Lee S Cha M. Design and optimization of rotating triboelectric nanogenerator by water electrification and inertia. Nano Energy. (2016) 27:340–51. 10.1016/j.nanoen.2016.06.051

[22] Fan K Wei D Zhang Y Wang P Tao K Yang R. A whirligig-inspired intermittent-contact triboelectric nanogenerator for efficient low-frequency vibration energy harvesting. Nano Energy. (2021) 90:106576. 10.1016/j.nanoen.2021.106576

[23] Park M Cho S Yun Y La M Park S Choi D. A highly sensitive magnetic configuration-based triboelectric nanogenerator for multidirectional vibration energy harvesting and self-powered environmental monitoring. Int J Energy Res. (2021) 45:18262–74. 10.1002/er.7003

[24] Ko H-J Kwon D-S Bae K Kim J. Self-suspended shell-based triboelectric nanogenerator for omnidirectional wind-energy harvesting. Nano Energy. (2022) 96:107062. 10.1016/j.nanoen.2022.107062

[25] Zhang C Zhou L Cheng P Liu D Zhang C Li X. (2021). Bifilar-pendulum-assisted multilayer-structured triboelectric nanogenerators for wave energy harvesting. Adv Energy Mater. 11:2003616. 10.1002/aenm.202003616

[26] Wang J Wang H Thakore N Lee C. Self-powereded direct muscle stimulation using a triboelectric nanogenerator (TENG) integrated with a flexible multiple-channel intramuscular electrode. ACS Nano. (2019) 13:3589–99. 10.1021/acsnano.9b00140 30875191

[27] Peng X Dong K Ning C Cheng R Yi J Zhang Y. All-nanofiber self-powereded skin-interfaced real-time respiratory monitoring system for obstructive sleep apnea-hypopnea syndrome diagnosing. Adv Funct Mater. (2021) 31:2103559. 10.1002/adfm.202103559

[28] Luo J Han K Wu X Cai H Jiang T Zhou H. Self-powered mobile sterilization and infection control system. Nano Energy. (2021) 88:106313. 10.1016/j.nanoen.2021.106313

[29] Tuells J Henao-Martínez AF Franco-Paredes C. Yellow fever: a perennial threat. Arch Med Res. (2022) 53:649–57. 10.1016/j.arcmed.2022.10.005 36404585

[30] Khongwichit S Chansaenroj J Chirathaworn C Poovorawan Y. Chikungunya virus infection: molecular biology, clinical characteristics, and epidemiology in Asian countries. J Biomed Sci. (2021) 28:84. 10.1186/s12929-021-00778-8 34857000 PMC8638460

[31] Harrington LC Edman JD Scott TW. Why do female Aedes aegypti (Diptera: Culicidae) feed preferentially and frequently on human blood? J Med Entomol. (2001) 38:411–22. 10.1603/0022-2585-38.3.411 11372967

[32] Muller DA Depelsenaire AC Young PR. Clinical laboratory diagnosis of dengue virus infection. J Infect Dis. (2017) 215:S89–S95. 10.1093/infdis/jiw649 28403441

[33] Onyango MG Ciota AT Kramer LD. The vector - host - pathogen interface: the next frontier in the battle against mosquito-borne viral diseases? Front Cell Infect Microbiol. (2020) 10:564518. 10.3389/fcimb.2020.564518 33178624 PMC7596266

[34] Sang R Lutomiah J Chepkorir E Tchouassi DP. Evolving dynamics of Aedes-borne diseases in Africa: a cause for concern. Curr Opin Insect Sci. (2022) 53:100958. 10.1016/j.cois.2022.100958 35878761

[35] Dengue and Severe Dengue. Available online at: https://www.who.int/zh/news-room/fact-sheets/detail/dengue-and-severe-dengue (accessed December 4, 2022).

[36] Milner DA. Malaria pathogenesis. Cold Spring Harb Perspect Med. (2018) 8:a025569. 10.1101/cshperspect.a025569 28533315 PMC5749143

[37] Malaria. Available online at: https://www.who.int/news-room/fact-sheets/detail/malaria (accessed December 1, 2022).

[38] Rubert A Guillon-Grammatico L Chandenier J Dimier-Poisson I Desoubeaux G. Insecticide resistance in Anopheles mosquitoes: additional obstacles in the battle against malaria. Med Sante Trop. (2016) 26:423–31. 10.1684/mst.2016.0634 28073732

[39] Lourens GB Ferrell DK. Lymphatic filariasis. Nurs Clin North Am. (2019) 54:181–92. 10.1016/j.cnur.2019.02.007 31027660

[40] Lymphatic Filariasis. Available online at: https://www.who.int/news-room/fact-sheets/detail/lymphatic-filariasis (accessed November 30, 2022).

[41] Sripadmanabhan Indira S Aravind Vaithilingam C Oruganti KSP Mohd F Rahman S. Nanogenerators as a sustainable power source: state of art, applications, and challenges. Nanomaterials. (2019) 9:773. 10.3390/nano9050773 31137520 PMC6566161

[42] Slabov V Kopyl S Santos M Kholkin AL. Natural and Eco-Friendly Materials for Triboelectric Energy Harvesting. “*Nano-Micro Letters*.”. (2020) 12:183–200. 10.1007/s40820-020-0373-y 34138259 PMC7770886

[43] Dudem B Kim D Mule A Yu J. Enhanced performance of microarchitectured PTFE-based triboelectric nanogenerator via simple thermal imprinting lithography for self-powereded electronics. ACS Appl Materials Interf. (2018) 10:24181–92. 10.1021/acsami.8b06295 29947215

[44] Wen RM Guo JM Yu AF Zhang K. Remarkably enhanced triboelectric nanogenerator based on flexible and transparent monolayer titania nanocomposite. Nano Energy. (2018) 50:140–7. 10.1016/j.nanoen.2018.05.037

[45] Niu Q Huang L Lv S Shao H Fan S Zhang Y. Pulse-driven bio-triboelectric nanogenerator based on silk nanoribbons. Nano Energy. 74:104837. 10.1016/j.nanoen.2020.104837

[46] Luo J Wang ZL Recent progress of triboelectric nanogenerators: from fundamental theory to practical applications. EcoMat. (2020) 2:e12059. 10.1002/eom2.12059

[47] Lei R Shi Y Ding Y Nie J Li S Wang F. Sustainable high-voltage source based on triboelectric nanogenerator with a charge accumulation strategy. Energy Environ Sci. (2020) 13:2178–90. 10.1039/D0EE01236J

[48] Meng K Chen J Li X Wu Y Fan W Zhou Z. Flexible weaving constructed self-powered pressure sensor enabling continuous diagnosis of cardiovascular disease and measurement of cuffless blood pressure. Adv Func Mat. (2019) 29:1806388. 10.1002/adfm.201806388

[49] He L Zhang C Zhang B Yang Q Yuan W Zhou L. A dual-mode triboelectric nanogenerator for wind energy harvesting and self-powered wind speed monitoring. ACS Nano. (2022) 16:6244–54. 10.1021/acsnano.1c11658 35312283

[50] Li J Kang L Long Y Wei H Yu Y Wang Y. Implanted battery-free direct-current micro-power supply from in vivo breath energy harvesting. ACS Appl.Mater Interfaces. (2018) 10:42030–8. 10.1021/acsami.8b15619 30444344 PMC6456428

[51] Kim J Lee J Go TW Rajabi-Abhari A Mahato M Park J. Skin-attachable and biofriendly chitosan-diatom triboelectric nanogenerator. Nano Energy. (2020) 75:104904. 10.1016/j.nanoen.2020.104904

[52] Wang J Xia K Liu J Li T Zhao X Shu B. Self-powered silicon PIN photoelectric detection system based on triboelectric nanogenerator. Nano Energy. (2020) 9:104461. 10.1016/j.nanoen.2020.104461

[53] Yoon HJ Kang M Seung W Kwak SS Kim J Kim HT. Microdischarge-based direct current triboelectric nanogenerator via accumulation of triboelectric charge in atmospheric condition. Adv Energy Mater. (2020) 10:2000730. 10.1002/aenm.202000730

[54] Liu D Zhou L Wang ZL Wang J. Triboelectric nanogenerator: from alternating current to direct current. iScience. (2021) 102018. 10.1016/j.isci.2020.102018 33490924 PMC7809521

[55] Price B Baker E. Nightlife: a cheap, robust, led based light trap for collecting aquatic insects in remote areas. Biodiv Data J. (2016) 4:e7648. 10.3897/BDJ.4.e7648 27099554 PMC4822069

